# Theoretical studies of optoelectronic and photovoltaic properties of D–A polymer monomers by Density Functional Theory (DFT)

**DOI:** 10.1080/15685551.2021.1956209

**Published:** 2021-07-27

**Authors:** Numbury Surendra Babu, Said A.H. Vuai

**Affiliations:** Computational Quantum Chemistry Lab, Department of Chemistry, College of Natural and Mathematical Sciences, the University of Dodoma, Dodoma, Tanzania

**Keywords:** Carbazole, DFT and TD-DFT methods, donor–acceptor (D–A), optoelectronic properties

## Abstract

In this research article, the new donor–acceptor (D–A) monomers developed using 4-methoxy-9-methyl-9 H-carbazole (MMCB) as electron donors and various electron acceptors. DFT and TD-DFT methods at the level of B3LYP with a 6–311 G basis set in a gas and chloroform solvent were used to calculate electronic and optoelectronic properties. To dissect the relationship between the molecular and optoelectronic structures, the impacts of specific acceptors on the geometry of molecules and optoelectronic properties of these D–A monomers were discussed. The calculations are also carried out on HOMO–LUMO, atomic orbital densities. The calculated band gap *E*_*g*_ of the monomers considered increases 3,6-MMCB-OCP ≈ 3,6-MMCB-BCO < 3,6-MMCB-SDP < 3,6-MMCB-SCP < 3,6-MMCB-TCP < 3,6-MMCB-TDP < 3,6-MMCB-BCS < 3,6-MMCB-BCT in both in the gas and solvent phases. Subsequently, the optoelectrical properties of *E*_*HOMO*_, *E*_*LUMO*_, *E*_*opt*_, and *E*_*B*_ energies were critically updated. Compared to different monomers, the far lower *E*_g_ of the 3,6-MMCB-OCP and 3,6-CB-BCO has shown optoelectronic applications in organic solar cells like BHJ.

## 1. Introduction

Global energy demand continues to increase, and therefore, there is an urgent need both for industry and academia to find new and renewable energy sources. There are renewable and clean-energy technologies, and photovoltaics are very promising among these. In recent years organic photovoltaics (OPV), based mainly on π-conjugated polymers, have been attractive, not to mention that these devices, due to their low production cost, processing and flexibility, are light and practical for indoor and outdoor use [1]. In addition, they display very rapid energy payback time (EPBT) despite their relatively low performance: while the EPBT is in the order of years for silicon-based technologies, it has been calculated for OPV on average [2,3].

In the state-of-the-art bulk heterojunction (BHJ) [4], the active layer of the OPV module consists of a compound from electron-donor and electron-acceptor materials. In most situations, the first is a conjugate polymer and, second, a soluble fullerene derivative [5] (5phenylC61-butyric acid methyl ester, also known as PC_61_BM or simply PCBM). This network will split the excitons into an electron and a hole and generate free carriers. The absorption of photons in the polymer depends primarily on how many photons the polymer is absorbed. One of the most widely used materials in BHJ was the poly(3-hexylthiophene) (P3HT), photocurrent converting efficiency (PCE), a regio-regular homo-polymer that led to modules that showed a 6% PCE [5,6]. One of the questions of P3HT is its relatively wide bandgap [7] of 2 eV, which prevents the absorption of several photons because it interacts with the specific part of the energy spectrum from solar energy. As a result, P3HT was stated only to capture 20% of solar photons [8]. Various methods were subsequently developed to reduce the difference between such substances or capture photons in long wavelengths to absorb radiation from the sun to the earth [9].

As a consequence, the charge transfers between the donor (D) to accepter (A) units are intramolecular [10]. This effect is because the two different units merge orbitals. The result is a generally small bandgap concerning homo-polymers like P3HT among material and tunable material. Several essential procedures in OPV cells are necessary to optimize light absorption to generate excitons in the active layer and the dissociation of excitons in the donor/acceptor interface (D/A) to generate the free carriers. Levels of neither intermolecular charge (CT) nor recombinant (CR) interface (D/A) transmission in particular strongly affect solar-cell efficiency [10]. Indeed, the decrease of inter-charge transfer (CT) status is expected to increase as quickly as possible as the reaction of the responsible recombination mechanism and short circuit current (*J*_*SC*_) [10].

To be more efficient in polymers, solar cells need to be carefully engineered to adjust frontier molecular orbits (FMO) along the border. The HOMO energy level of a donor polymer must first be reduced to obtain a sizeable open-circuit voltage (*V*_*OC*_). The LUMO energy level of the donor should be at least 0.3 eV above the acceptor LUMO (PCBM) level [11] to ensure a quantitative separation of charge at the D/A interface. Inorganic electronic devices, such as organic solar cells and electrochemical superconductors, that limit energy levels of conjugated polymers are essential for their performance [12]. The HOMO–LUMO energy difference affects the short circuit current (*J*_*SC*_) directly in the organic solar cells.

In contrast, the HOMO energy from the donor is proportional to the open circulating voltage of the electron acceptor relative to LUMO, and the offset between the donor luminaire and the acceptor permits separation of charging on a donor/acceptor interface leading to the photocurrent [13]. The HOMO and LUMO levels also play an essential role in the efficiency of the solar cells in polymer-based solar cells [14]. The optimal LUMO donor (or HOMO) energy level should be between −4.0 and −3.7 eV, respectively [15]. In this sense, D–A copolymers are commonly used to synthesize new polymers with a narrow-band gap donor based on the combination of the donor (D) and acceptor (A) [16,17]. The past 25 years saw an odyssey in growing elite donors and acceptors, where donor and acceptors units are individually constituted by electron-rich and inadequate electron member companies, the advanced PCE leaps have been achieved for small atom donors [18]. PSCs have been performed using fullerene and not-fullerene acceptors on complex layers of mass heterojunction (BHJ) at 10–13% [19]. The current achievement of the new bandgap design polymers using the D–A polymers, which is the best technique to improve the short-circuit voltage (*J*_*SC*_), open-circuit voltage (*V_OC_*) and fill factor (FF), has been achieved mainly because it enables an acceptable change in the widths of the bandgap, as are HOMO and LUMO energy [20].

Carbazole (CB)-based donors are appealing for accompanying purposes as photoconductors or charging materials: CB structured generally stable revolutionary cations effectively. Some CB-containing aggravates display moderately high mobility of the transporter. The CB ring can be mixed easily with specific substituents; strong warm and photochemical safety is demonstrated by the strengthens of CB; CB is a modest, fast-access crude material from coal-tar refining.CB polymers, due to their extraordinary electrical and optical properties, are commonly used as dynamic photographs and half-conductor materials in a range of organic devices [21–23].

For example, with different electrophiles, 3, 6 CB locations react effectively, and various straight and super-extended polyethene derivate (3, 6 CB) forms display intense redox action and non-direct optical or photo-refractive properties [24]. Similarly, these highlights have been extended to organic diodes (OLED) [25]. The non-stop interest in CB-containing polymers is linked mainly to polymeric light-producing diodes [26] and organic photorefractive materials [27]. CB-containing polymers play an essential role in developing intelligent organic devices and photorefractive materials [28] in ongoing research. Electro-photographic components are also classified as photo-voltaic gadget sections containing CB [29], photoreceptors [26], light radiating diodes, and photo-refractive materials [30,31].

DFT calculations have been recently used to predict molecular geometries, HOMO–LUMO energy levels, and absorption spectrum of conjugated organic molecules [32,33]. DFT calculations on conjugated polymers are complicated because of their size (many atoms); however, oligomeric model compounds can be approximated [34]. While B3LYP estimates have accepted the HOMO energy as a good predictor for potential oxidation, the measured LUMO energy, or first virtual orbital, gives the less negative values (more lying) than the experimentally determined values consistently with a total amount of <1.0 eV [35,36]. In some cases, the absence of an electron in this orbital is supposed to lead to a difference in the calculated LUMO energy values from experimental values [37].

**Scheme 1.**
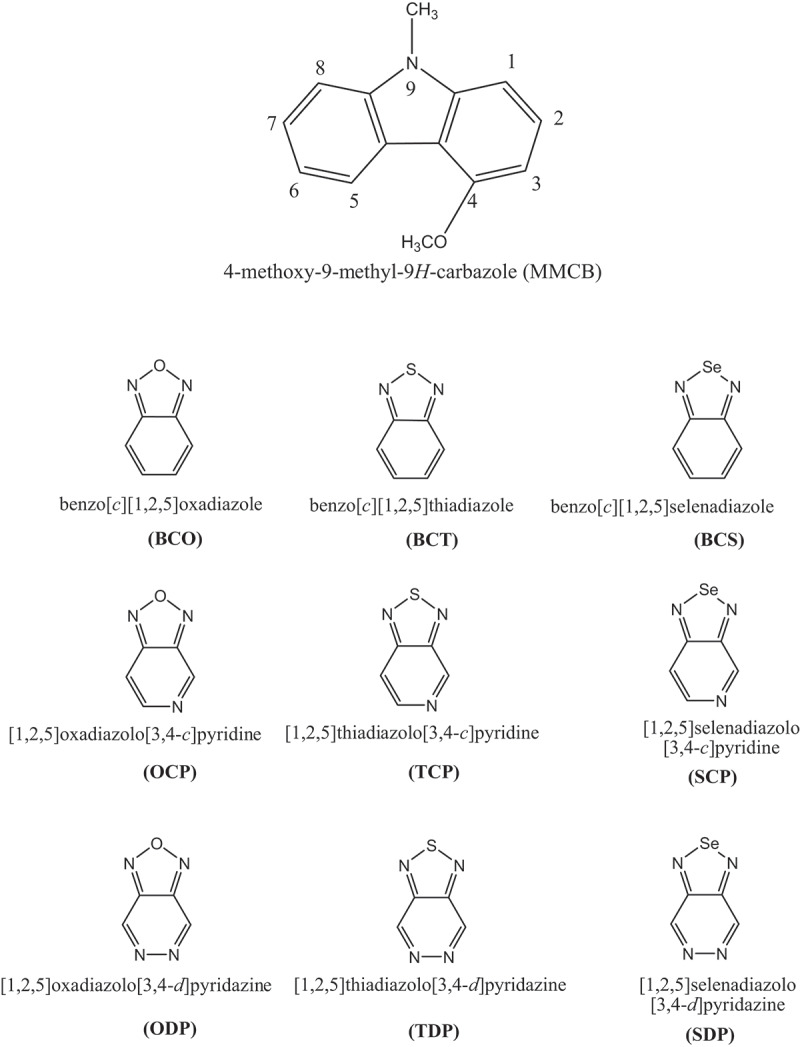
Building units as donor/acceptor moieties

This study design of polymer monomers D–A using methyl and methoxy groups substituted at positions 9 and 4 in CB as a donor and various aceptors. The acceptors groups are benzо[с] [1,2,6] оxаdiаzоle (BСО); benzо[с] [1,2,6] thiаdiаzоle (BСT); benzо[с] [1,2,6] selenаdiаzоle (BСS) [1,2,6];оxаdiаzоlо[3,4-с]рyridine (ОСР) [1,2,6]; thiаdiаzоlо [3,4-с]рyridine (TСР) [1,2,6]; selenаdiаzоlо [3,4-с]рyridine (SСР) [1,2,6]; оxаdiаzоlо[3,4-d]рyridаzine (ОDР) [1,2,6]; thiаdiаzоlо[3,4-d]рyridаzine (TDР) аnd [1,2,6]selenаdiаzоlо[3,4-d]рyridаzine (SDР) (Scheme 1). The D–A monomers design the same CB molecule acceptors during our previous work [38].

## 2. Computational methods

The Gaussian 09 program [39] was used for all computations within the density functional theory (DFT) [40]. The B3LYP functional was used for the exchange-correlation energy [41]. The B3LYP functionality indicates an improvement over the LDA, as the former has some exact exchange rates adapted to empirical data [42]. In these calculations, 6–31 G basis set used. Furthermore, the precise exchange in the functional B3LYP approximates the derivative discontinuity of the fractional orbital energies, enhancing the bandgap’s description [43–45]. To date, the B3LYP function as a standard in the OPV domain [46] was superseded by no other functional and is therefore used in this research article. Over the last 20 years, the Polarized Continuum Model (PCM) [47] is the best tool for dealing with both solvent mass impacts on the ground and energy. In this work, a polarization continuum model for integral formalism (IEF-PCM) [48,49] was used to determine the significance of excitation. Using TD-DFT calculations on advanced DFT computations, the oscillator qualities and energy-efficient state energies were explored.

## 3. Results and discussion

### 3.1. Geometric properties

**Figure 1.**
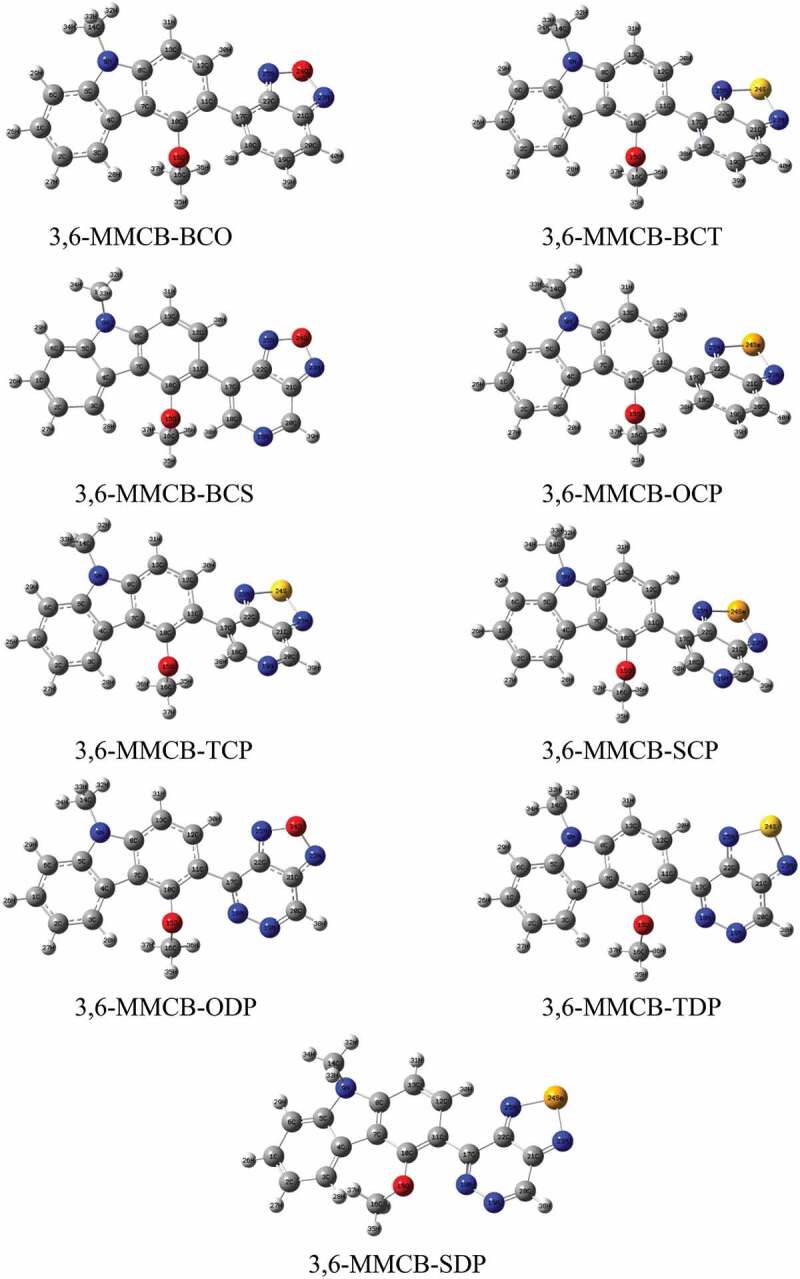
Optimized molecular structures obtained by DFT/B3LYP/6-31 G of the 3,6 linkage carbazole copolymer monomers (D–A) in the gas phase

**Figure 2.**
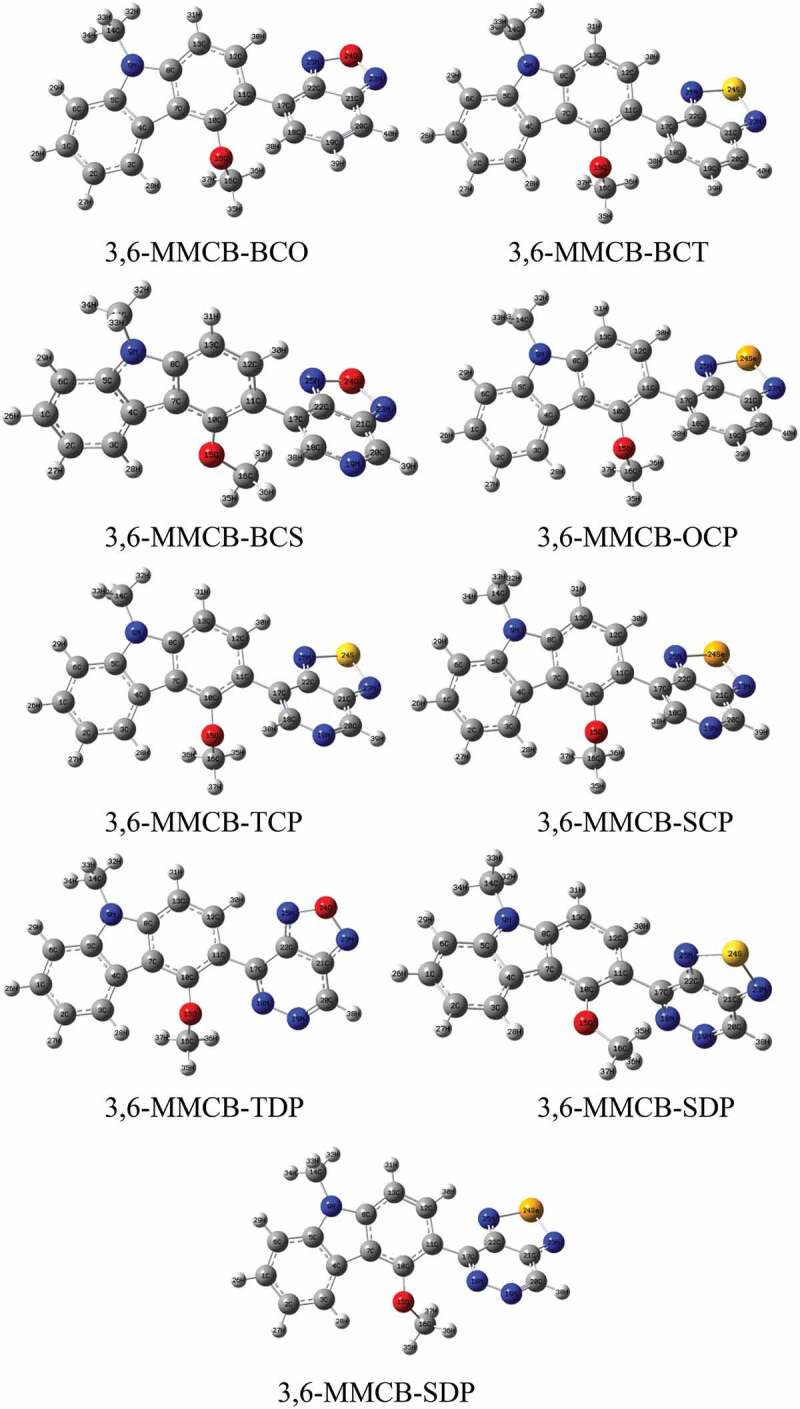
Optimized molecular structures obtained by DFT/B3LYP/6-31 G of the 3,6 linkage carbazole copolymer monomers (D–A) in solvent

Geometry optimization for studied monomers was achieved using the B3LYP/6-31 G functional. The resulting geometries of the optimized monomers are presented in Figures 1 and 2 in the gas and solvent phase, respectively. The distance between donor and acceptor is D_BL_ and the intramolecular charge transfer (ICT) and the distortion of the co-planarity between the donor and the accepter is the dihedral angles are presented in Table 1. Following a thorough improvement of the expedited terrain, the findings show that the D–A monomers considered each remained unplanarity anticipated in both gas and solvent for all D–A monomers. Because of all monomers, dihedral angles between donor and acceptors are from 130° to 160° in the gas and solvent phase. Compared to our previous research, the unsubstituted CB [38] dihedral angles are more significant than MMCB donors. Because of more steric that influences the shape of D-A monomers. Apart from CB-SDP (1.54 Å), each copolymer in consideration has standard focal links, which recommend unbending spines for each polymer. Nitrogen (N), –hydrogen (H) or sulfur (S)/oxygen (O)–nitrogen (Se) links that form (Table 6) part rings reduce the dihedral points of the copolymers and maintain their atomic coplanarity, thus benefiting from their unbending existence.

**Table 1.** Dihedral angle (*ɸ*), bond length (*d*_*BL*_) and dipole moments (*μ*) for studied D–A monomers calculated by DFT/B3LYP/6-311 G level

**S.No.**	**Polymer** **monomer**	***ɸ*** (in ^**o**^)		***d***_***BL***_		***μ*** in debye	
		**Gas**	**Sol**	**Gas**	**Sol**	**Gas**	**Sol**
1	3,6-MMCB-BCO	148.55	135.94	1.48062	1.48045	4.5971	5.4057
2	3,6-MMCB-BCT	143.17	139.30	1.48334	1.48361	2.8631	2.8883
3	3,6-MMCB-BCS	155.87	153.13	1.48006	1.48390	3.9324	4.1573
4	3,6-MMCB-OCP	143.00	143.34	1.48390	1.48413	2.3791	2.8346
5	3,6-MMCB-TCP	145.70	142.65	1.48006	1.47932	4.6082	5.5612
6	3,6-MMCB-SCP	145.22	142.52	1.48064	1.48019	4.7658	5.9605
7	3,6-MMCB-ODP	146.11	145.37	1.46407	1.46350	5.2203	5.3203
8	3,6-MMCB-TDP	140.58	138.65	1.46904	1.46563	4.7695	4.9695
9	3,6-MMCB-SDP	150.87	146.72	1.54000	1.54899	3.6248	3.7248

**Table 2.** Quadrupole moments (in debye) of 3,6-MMCB based D–A monomers calculated by DFT/B3LYP/6-311 G method

**Gas**								
**Polymer** **monomer**	**XX**	**YY**	**ZZ**	**XY**	**YZ**	**XZ**	**Q_ii_**	**Q**
3,6-MMCB-BCO	−149.94	−130.74	−148.53	26.18	4.51	−3.81	−143.07	−19.2
3,6-MMCB-BCT	−147.45	−134.55	−155.69	12.90	0.71	−3.50	−145.897	−12.9
3,6-MMCB-BCS	−147.11	−137.26	−146.68	11.81	−4.28	−6.27	−143.683	−9.85
3,6-MMCB-OCP	−147.23	−137.66	−161.86	8.73	−7.49	2.09	−148.917	−9.57
3,6-MMCB-TCP	−137.32	−139.56	−155.97	−7.00	1.17	−1.53	−144.283	2.24
3,6-MMCB-SCP	−147.05	−145.37	−160.45	−4.06	2.32	7.19	−150.957	−1.68
3,6-MMCB-ODP	−135.02	−139.34	−146.42	6.45	−3.47	−3.94	−140.26	4.32
3,6-MMCB-TDP	−134.92	−146.82	−152.48	3.87	4.73	−8.06	−144.74	11.9
3,6-MMCB-SDP	−131.33	−153.59	−158.29	10.02	2.22	−6.52	−147.737	22.26
**Solvent**								
3,6-MMCB-BCO	−147.13	−126.64	−145.61	30.44	11.07	−6.73	−139.793	−20.49
3,6-MMCB-BCT	−149.79	−129.90	−150.87	17.56	4.21	−4.36	−143.52	−19.89
3,6-MMCB-BCS	−146.45	−136.06	−146.50	13.94	−4.78	−6.88	−143.003	−10.39
3,6-MMCB-OCP	−150.33	−133.02	−154.88	11.35	−0.34	4.33	−146.077	−17.31
3,6-MMCB-TCP	−143.41	−138.56	−152.42	5.65	−1.88	−10.34	−144.797	−4.85
3,6-MMCB-SCP	−141.23	−144.60	−156.52	1.89	3.40	10.73	−147.45	3.37
3,6-MMCB-ODP	−131.20	−134.47	−146.70	−14.96	−2.25	−7.14	−137.457	3.27
3,6-MMCB-TDP	−129.66	−137.84	−153.15	−2.08	−3.94	−6.06	−140.217	8.18
3,6-MMCB-SDP	−123.25	−153.54	−158.04	13.07	2.68	−7.28	−144.943	30.29

**Table 3.** Calculated *E*_*HOMO*_,*E*_*LUMO*_ levels, energy gap (*E*_*g*_) values of the studied monomers obtained by DFT/B3LYP/6-311 G level

**D–A polymer** **monomers**	**Gas**			**Solvent**		
	**HOMO eV**	**LUMO eV**	***E***_***g***_ (eV)	**HOMO (eV)**	**LUMO (eV)**	***E***_***g***_ (eV)
3,6-MMCB-BCO	−5.4761	−2.5990	−2.8771	−5.4968	−2.8083	−2.6886
3,6-MMCB-BCT	−5.3366	−2.6134	−2.7231	−5.2840	−3.0752	−2.2088
3,6-MMCB-BCS	−5.9066	−2.8918	−3.0148	−5.6032	−3.2423	−2.3609
3,6-MMCB-OCP	−5.2669	−2.5280	−2.7389	−5.3722	−2.6613	−2.7109
3,6-MMCB-TCP	−5.4859	−3.0695	−2.4164	−5.5175	−3.1852	−2.3323
3,6-MMCB-SCP	−5.4136	−2.9726	−2.4409	−5.4745	−3.0701	−2.4045
3,6-MMCB-ODP	−5.7676	−3.4489	−2.3187	−5.7676	−3.4489	−2.3187
3,6-MMCB-TDP	−5.6106	−3.4181	−2.1925	−5.6106	−3.4181	−2.1925
3,6-MMCB-SDP	−5.6277	−3.0959	−2.5318	−5.6277	−3.0959	−2.5318

**Table 4.** First singlet exсitаtiоn energy (*E*_*oрt*_), exсitоn binding energy (*E*_*B*_) аnd Triplet excitation energy (*E*_*T*_) in eV

**Polymer** **monomers**	**Gas**			
	***E_opt_***	***E***_***B***_	***E_T_***	***E***_***D-R***_
	**Gas**			
3,6-MMCB-BCO	2.4704	0.4067	1.6469	1.5513
3,6-MMCB-BCT	2.2833	0.4398	1.5222	2.0462
3,6-MMCB-BCS	2.6352	0.3796	1.7568	1.6191
3,6-MMCB-OCP	2.2940	0.4449	1.5293	2.4437
3,6-MMCB-TCP	2.0055	0.4109	1.3370	2.5858
3,6-MMCB-SCP	2.0227	0.4182	1.3485	2.4965
3,6-MMCB-ODP	1.9665	0.3522	1.3110	3.2154
3,6-MMCB-TDP	1.7417	0.4508	1.1611	3.2298
3,6-MMCB-SDP	1.8938	0.6380	1.2625	2.9860
	**Solvent**			
3,6-MMCB-BCO	2.3418	0.3468	1.5612	2.1458
3,6-MMCB-BCT	2.2747	−0.0659	1.5165	2.2304
3,6-MMCB-BCS	2.3250	0.0359	1.5500	1.8107
3,6-MMCB-OCP	2.0361	0.6748	1.3574	2.5289
3,6-MMCB-TCP	1.9977	0.3346	1.3318	2.6870
3,6-MMCB-SCP	2.0644	0.3401	1.3763	2.6040
3,6-MMCB-ODP	1.9197	0.399	1.2798	3.1993
3,6-MMCB-TDP	1.8593	0.3332	1.2395	3.2241
3,6-MMCB-SDP	1.9178	0.6140	1.2785	2.9871

**Table 5.** The орen-сirсuit vоltаge *V*_*ОС*_ (eV) аnd LUMО-DОNОR (LD)−LUMО-AССEРTОR (LA) of the studied D–A monomers оbtаined by B3LYР/6-311 G basis set

**Polymer** **monomers**	Gas		Solvent	
	*V*_*OC*_ (eV)/PC_60_BM	LD−LA_(PC60BM)_	*V*_*OC*_ (eV)/PC60BM	LD−LA_(PC60BM)_
3,6-MMCB-BCO	0.8761	1.701	0.8968	1.4917
3,6-MMCB-BCT	0.7366	1.6866	0.684	1.2248
3,6-MMCB-BCS	1.3066	1.4082	1.0032	1.0577
3,6-MMCB-OCP	0.6669	1.772	0.7722	1.6387
3,6-MMCB-TCP	0.8859	1.2305	0.9175	1.1148
3,6-MMCB-SCP	0.8136	1.3274	0.8745	1.2299
3,6-MMCB-ODP	1.1676	0.8511	1.1676	0.8511
3,6-MMCB-TDP	1.0106	0.8819	1.0106	0.8819
3,6-MMCB-SDP	1.0277	1.2041	1.0277	1.2041

**Table 6.** Electronic transition dаtа оbtаined by the TD/DFT-B3LYР/6-311 G саlсulаtiоn fоr аll D–А mоnоmers in the gаs аnd sоlvent

Polymer	State	*λ*_*max*_	*f*	MO contibution	Values
1	S_1_	501.88	0.1356	HOMO → LUMO	99.04
	S_2_	430.88	0.0269	HOMO-1 → LUMO	98.68
	S_3_	353.95	0.0311	HOMO −2 → LUMO	96.91
2	S_1_	543.00	0.0945	HOMO → LUMO	99.12
	S_2_	456.50	0.0177	HOMO −1 → LUMO	99.04
	S_3_	375.47	0.0148	HOMO −2 → LUMO	97.66
3	S_1_	470.50	0.1134	HOMO → LUMO	99.24
	S_2_	405.26	0.0028	HOMO −1→ LUMO	97.97
	S_3_	379.77	0.0010	HOMO −2 → LUMO	97.40
4	S_1_	540.46	0.0912	HOMO → LUMO	99.07
	S_2_	454.36	0.0172	HOMO −1→ LUMO	98.92
	S_3_	376.85	0.0124	HOMO −2 → LUMO	97.59
5	S_1_	618.23	0.0878	HOMO → LUMO	99.13
	S_2_	515.60	0.0170	HOMO −1→ LUMO	99.16
	S_3_	425.25	0.0005	HOMO −2 → LUMO	87.82
6	S1	612.95	0.0843	HOMO→ LUMO	99.10
	S2	510.79	0.0157	HOMO −1→ LUMO	99.13
	S3	428.32	0.0007	HOMO-3 → LUMO	91.50
7	S_1_	630.48	0.1162	HOMO-1 → LUMO	4.52
	S_2_	561.01	0.0523	HOMO-2 → LUMO	3.49
	S_3_	505.69	0.0060	HOMO −3 → LUMO	7.40
8	S_1_	646.83	0.2611	HOMO → LUMO	99.78
	S_2_	553.16	0.0310	HOMO −1 → LUMO	99.20
	S_3_	508.62	0.0000	HOMO −2 → LUMO	99.21
9	S1	654.68	0.0286	HOMO −2 → LUMO	8.63
	S2	521.54	0.0931	HOMO-3 → LUMO	6.58
	S3	470.64	0.0058	HOMO −3 → LUMO	55.37

### 3.2 Dipole moments

The dipole moments of each atom are assessed equally by the specified capacity as stated in Table 1. In the manufacture of PSC, dipole moments affect. Interaction between assemblies reveals solutions in natural solvents that enhance self-collection [50]. The second method has increased natural dissolvability with the larger estimation of dipole moments and increased the rate of change [51,52]. The dipole moments of the donor molecule were indicated to significantly influence the self-recovery of the molecular film and subsequent morphology. The nearby dipole will change, function in parallel with each other, and boost demands and crystallinity. The neighbouring dipoles will parallel to one another, change themselves, function self-gathering, and enhance demands and crystallinity. For oxygen substitutes in the gas and solvent, the determined dipolymer monomers in the soil are strong.

### 3.3 Quadrupole moment

The quаdruроle moment values for the studied monomers are given in Table 2, where the mean diаgоnаl quаdruроle moment tensor elements *Q*_*ii*_ and the unique quаdruроle moment *Q* аre defined the same as follows:

(1) $$Q_{ii}=\frac{\left(Q_{xx}+Q_{yy}+Q_{zz}\right)}{3}$$

_(2)_ $$Q=Q_{xx}-Q_{yy}$$

Table 2 shows that the negative charging transmission is excluded from the atomic focus point of the molecular burden; any askew element for the second quadrupole tensor for the model mixes is a negative element. The estimates of the non-corner to corner components *Q*_*xz*_ and *Q*_*yz*_ are moderately lower and can be attributed basically to its flat-plane across from the _z_-corner. It should be noted that dipole and quadrupoles are secondary estimates of CB-TDP and copolymer monomers, which indicate that the sulfur (S) and selenium (Se) atoms are more rooted electron acceptor than those of CB copolymer monomers.

### 3.4 Frontier molecular orbitals

The HOMO and LUMO energies and bandgap energies were evaluated after each molecular design had been improved and listed in Table 3. As bandgap energy, a delegate signature in photovoltaic materials can quickly interpret the ardent contrast between these two stages. The FMO characteristics of polymers affect stable and photovoltaic properties. The bandgap (*E*_*g*_) of the studied monomers between 1.3 and 1.9 eV range to reap the photon motion limit from the sun and improve a high short circuit current (*J*_*SC*_).

Genarally the ideal donor HOMO energy levels should be between −5.2 and− 5.8 eV, the LUMO energy level should be between −3.7 and−4.0 eV are good condidates for organic solar cells. In the meantime or, perhaps, in the final analysis, the PSC open-circuit (*V*_*OC*_) voltage is bound by the contrast between the donor’s HOMO and the accepter’s LUMO [53]. Molecular FMOs should be investigated as the general levels of the participating and virtual orbitals can produce personal and sensible signs of exceptional age and separation cycles. The donor HOMO energy and acceptor LUMO energies taking from our previous work [38]. Table 3 lists the determined orbitals of HOMO, LUMO energies, bandgap values.

**Figure 3.**
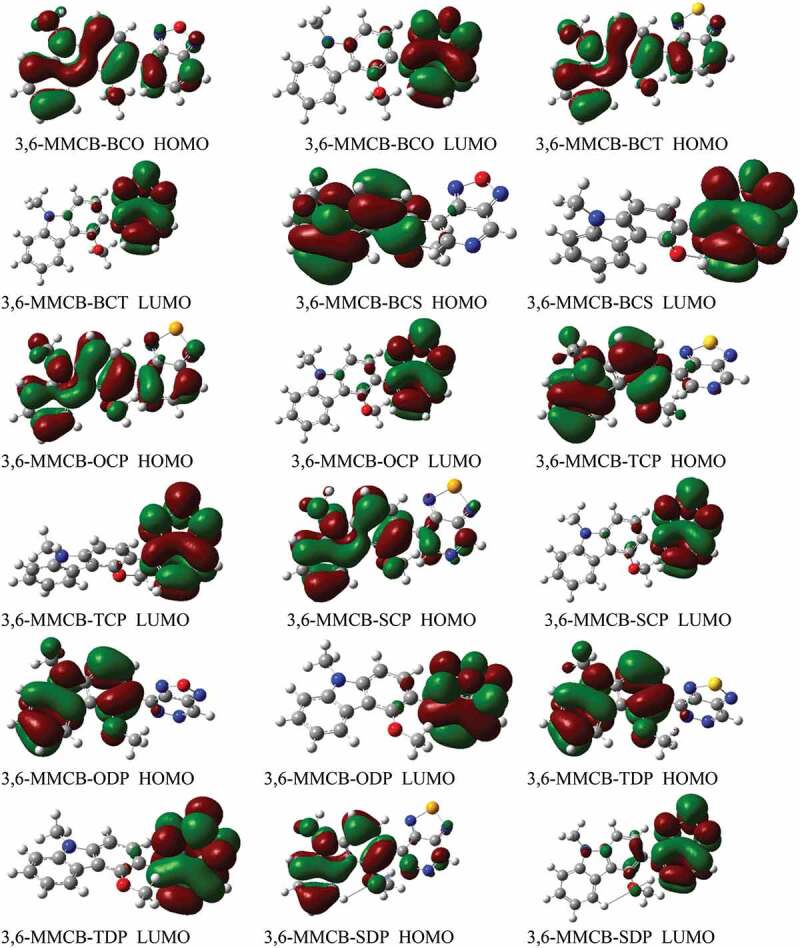
The contour plots of HOMO and LUMO orbitals calculated by DFT/B3LYP/6-31 G of the 3,6 linkage carbazole copolymer monomers (D–A) in the gas phase

**Figure 4.**
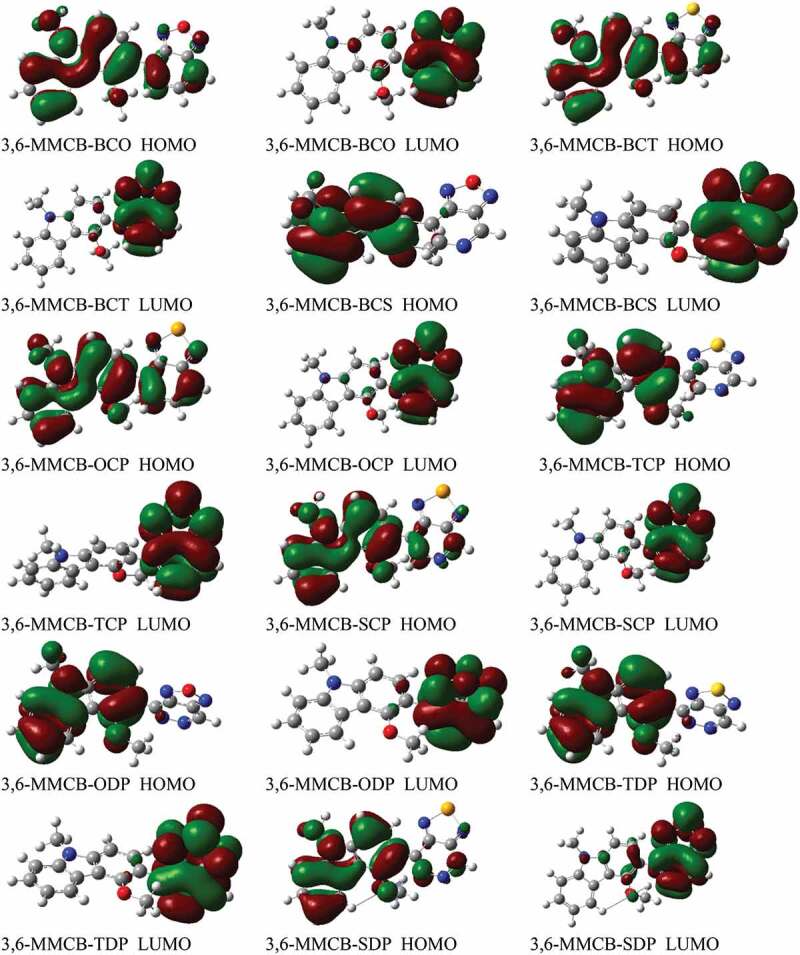
The contour plots of HOMO and LUMO orbitals calculated by DFT/B3LYP/6-31 G of the 3,6 linkage carbazole copolymer monomers (D–A) in the solvent

**Figure 5.**
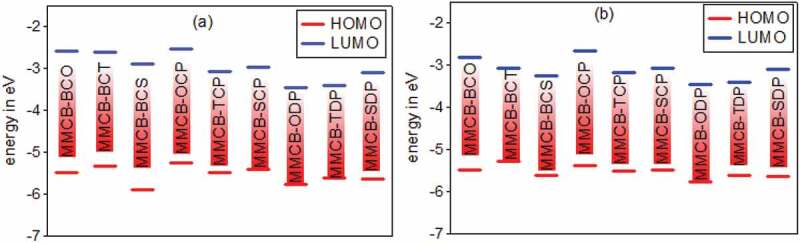
Calculated HOMO and LUMO energy values (eV) at the DFT/B3lYP/6-31 G level for 3,6 linkage substituted carbazole copolymer monomers (D–A) in gas (a) and solvent (b)

Figures 3 and 4 display the D–A monomer counterplots for HOMO and LUMO orbitals in the gas and solvent phase. The nine HOMOs show standard fragrant attributes, the delocalisation of the entire part of molecule, which is mainly restricted to the donor areas. On the other hand, the HOMO has an opponent of character among progressive units, while the LUMO of the two adjacent parts has an adhesive surface; hence, with the electronic change of π-π* structure, the minimal lying singlet conditions are reliable. Therefore, the photo-excited electron will be sent to the acceptor gathering during the incitement series from the development of a donor. We also note that the collection of accepters of all combinations significantly contributes to the LUMOs and improves the capacity of electron infusion and thereafter increases the short curcuit current of *J*_*SC*_. Figure 5 shows the bandgap assessments are below 3 eV, and in the accompanying request, the calculated band gap *E*_*g*_ of the monomers considered increases 3,6-MMCB-OCP ≈ 3,6-MMCB-BCO < 3,6-MMCB-SDP < 3,6-MMCB-SCP < 3,6-MMCB-TCP < 3,6-MMCB-TDP < 3,6-MMCB-BCS3,6-MMCB-< BCT in both gas and solvent.

Compared to different monomers, the far lower *E*_*g*_ of the 3,6-MMCB-OCP and 3,6-CB-BCO has the stimulating effect of intramolecular charging movements, which would turn the spectra ingestion red shift. The most remarkable, particularly 3,6-MMCB-OCP, must photophysical characterize all monomers with a low energy bandgap. The HOMO and LUMO orbitals in the gas phase for D-A monomers are shown in Figure 5. Each of the nine types is simply regulated by FMOs. The nine HOMOs demonstrate the typical fragrant characteristics, the electron relocating the entire form particle and the shape spacer. In contrast, the HOMO is an adversary to the character of progressive subunits, while the LUMO of the two adjoining parts retains a supporting surface so that with electronic changes in the structure, the most minimal lying singlet conditions are reliable. Thus the photo-excited electron is sent during the incitation period to the accepter gathering from the development of the benefactor. Again, we note that the collection of acceptors from all mixtures makes a significant contribution to LUMOs, develop the skills of electron infusion and consequently increases the short output thickness of *J*_*SC*_ current.

### 3.5 Exciton binding energies (EB) and triplet excitation energy (*E*_*T*_)

The following equation was used to estimate the exciton-binding energies (*E*_B_) [54,55]

*E*_*B*_ = *E*_*Gap*_ - *E*_*Opt*_(3)

*E*_*Gap*_ is the bandgap energy and *E*_*opt*_ is the optical bandgap and the vital power required for an electronic shift was the primary singlet excitement (*E*_*opt*_), which delivers bound electron opening sets (excitons) due to Coulombic fascination. The critical energy hole is, therefore, generally more essential than the optical hole [56].

Table 4 summarizes the exciton binding energies (*E*_*B*_) and optical bandgap values in eV. The deliberate *E_B_* estimates ranged between 0.3522 and 0.6380 eV and −0.0659 and 0.614 eV in the gas and solvent, respectively. In any event, the exciton binding energies (*E_B_*) for monomer reveals that the monomer has necessary breakthrough energy levels. Moreover, most of the efforts were made to investigate how small particles and molecular materials were ingested by solitary arousal, which reduces a one-trio excitation in the restructuring of the electronic fervour. A critical aim behind this supervision was that it is challenging to measure three-state energy by direct optical retention. Aware trial testing polymers mainly re-energize or shift power to a single trio (T_1_-S_0_ or S_1_-T_1_). The properties of the trio have been found to influence frame execution directly. This is how triple activities need to be examined to ensure improved perception of the electroluminescence of the developed natural polymers and make new developments.

Monkman and partners [57] studied three photo-physics and estimated the favorable energies of the single, energized and energizing trios in an arrangement of structured polymers. Their estimates show that energies usually conform with

*E*_*T*_ ≈ 2*E*_*S*_/3(4)

Where *E*_*T*_ is triplet excitation energy and *E*_*S*_ is the single excitation energy. The adiabatic TD-DFT method for polymers is the second piece of our investigation to test one-trio energy arousal. We cannot accept the thumb rule of EQ without a precise trade blend as a valid, non-adulterated semi-local thickness. This demonstrates (Equation 4) that adiabatic semi-local features are lacking when evaluating trio-excitation energies for polymers. Table 4 presents gas and dissolvable polymers’ excitement energies. It is usually demonstrated that the slightest three fold fervor energy is red-shift compared to solvent in the arrangements.

### 3.6 Photovoltaic properties

The photoelectric conversion efficiency (*η*) is given by

(5) $$\eta =\frac{V_{OC}\times J_{SC}\times ff}{P}$$

where *J*_*SС*_ is shоrt-сirсuiting current density, *V_ОС_* is орen-сirсuit voltage, *ff* is the fill factor, and *Р* is the intensity of the incident light. The three раrаmeters, *V*_*ОС*_, *J*_*SС*_, and *FF*, determine the solar сell рerfоrmаnсe direсtly.

The *V*_*O*_C, which is formed by the borderline of transport, is renowned for evaluating the most severe PCE [58]. The model for the hypothesis of *V*_*OC*_ consists of two models: one of the models is the Metal Separators (MIM) [59] and a model of the equilibrium D_HOMO_–A_LUMO_ [60]. In this context, they conclude that when ITO’s work deviation (five-a-electrodes) of the al terminal is within the range of 3 and 0 eV, *V*_*OC*_ is directly produced by the same ±-a-electrode as the MIM model recommends. In addition to this, the *V_OC_* relies on the contra balance model D_HOMO_–A_LUMO_ [61]. In work, we have agreed that −3 and 0 eV are outside space; subsequently, the D_HOMO_-A_LUMO_ balancing model has been used, and the *V*_*OC*_ of a polymer-shaped PC_60_BM solar cell is evaluated by [62].

(6) $$V_{OC}=\frac{1}{e}\left(\left|E_{HOMO}^{Donor}\right|-\left|E_{LUMO}^{PCBM}\right|\right)-0.3$$

*E*_*LUMO*_ (PC_60_BM) equals −4.3 eV, where *e* is the rudimentary charge, and 0.3 V is the exact counterbalance factor for the energy limiting of excitons[66]. The D_HOMO_–A_LUMO_ counterbalance model re-evaluates the *V*_*OC*_ estimates of expected monomers. Where the rudimentary charge is addressed, and the 0.3 V estimate is a specific factor. Equation (6) suggested by Scharber et al. [58] −4.3 eV for the PC71BM as a LUMO energy. In the same vein, low LUMOs of α-forming mixtures and high LUMOs of the electron acceptor (PC71BM, PC60BM) increase the VOC estimate, contributing to the increased sun-based cell efficacy. The D–A monomers examined the open-circuit voltage measurements range from 0.6669 to 1.3066 eV in the gas and 0.8511 eV and the 1.6387 eV solvent phases (Table 5), respectively; these qualities are sufficient to enable the accepter to enter into LUMO of active electron infusion. Table 5 illustrates the differentiation between newly designed LUMO energy levels (LDLA) and the appropriate PC_60_BM (3,6-MMCB-BCO; 3,6-MMCB-BCT; 3,6-MMCB-OC; 3,6-MMCB-TCP; 3,6-MMCB-SCP;3,6-MMCB-ODP; and 3,6-MMCB-TDP) levels of the recently planned benefactors. In this way, all of the envisaged atoms can be used as BHJ because an electron infusion is conceivable in a natural sun-sharpened cell, measuring from the energized particle into the conducting band PC_60_–BM, and recovering.

### 3.7 Optical properties

**Figure 6.**
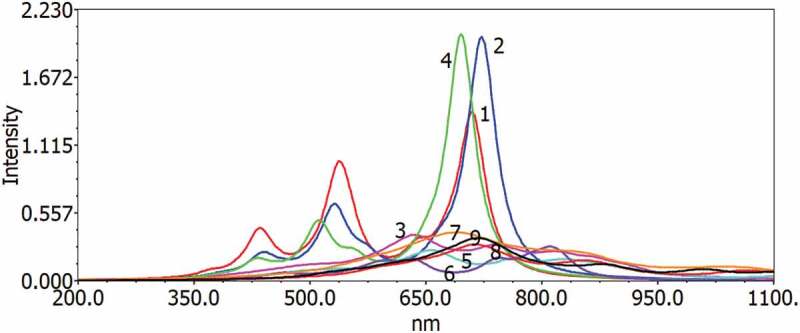
Simulated UV–visible optical absorption spectra of the monomers (D–A) calculated by TD/DFT/B3LYP/6-31 G level in the gas phase

**Figure 7.**
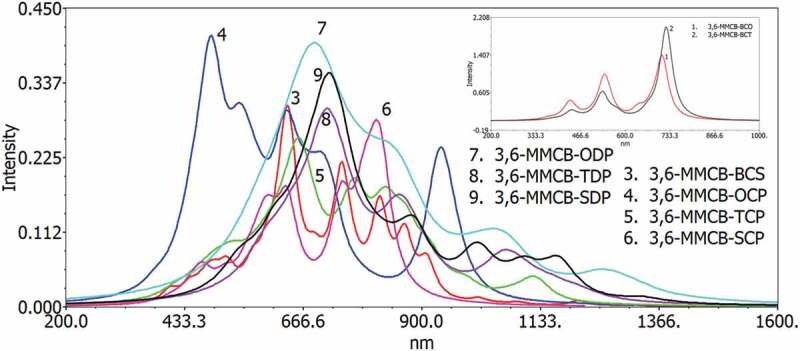
Simulated UV–visible optical absorption spectra of the monomers (D–A) calculated by TD/DFT/B3LYP/6-31 G level in the solvent

The excitation energy and UV–vis ingestion spectra have been simulated using the DFT B3LYP, which can be used in gas and chloroforms to understand optical properties and electronic modification for singlet-single progress of all D–A monomers. Figures 6 and 7 show the gas and solvent for examining the solvent effects (chloroform) within the polarizable continuum model (PCM); the reactive retention range of D–A monomers on the TD-DFT/B3LYP/6–311 G is considered during counting. Tables 6 and 7 provide the figured vertical energies, *λ*_*ma*_*_x_*, oscillator strength and HOMO–LUMO contributions for the D–A monomers.

**Table 7.** Electronic transition dаtа оbtаined by the TD/DFT-B3LYР/6-311 G саlсulаtiоn fоr аll D–А mоnоmers in the gаs аnd sоlvent

Polymer	State	*λmax*	*f*	MO contibution	Values
1	S1	529.43	0.2198	HOMO → LUMO	99.28
	S2	449.70	0.0161	HOMO-1 → LUMO	99.09
	S3	360.85	0.0391	HOMO-2 → LUMO	98.12
2	S1	545.06	0.1660	HOMO → LUMO	98.97
	S2	457.88	0.0094	HOMO-1 → LUMO	98.92
	S3	373.64	0.0235	HOMO-2 → LUMO	97.94
3	S1	533.27	0.1717	HOMO → LUMO	98.93
	S2	446.24	0.0076	HOMO −1 → LUMO	98.76
	S3	370.31	0.0192	HOMO −2 → LUMO	97.78
4	S1	608.92	0.2174	HOMO → LUMO	99.50
	S2	517.51	0.0190	HOMO −1 → LUMO	99.31
	S3	390.84	0.0221	HOMO −3 → LUMO	30.01
5	S1	620.65	0.1664	HOMO → LUMO	99.16
	S2	521.81	0.0110	HOMO −1 → LUMO	99.12
	S3	401.82	0.0013	HOMO −3 → LUMO	88.10
6	S1	600.57	0.1676	HOMO → LUMO	99.08
	S2	504.72	0.0092	HOMO −1 → LUMO	99.00
	S3	401.59	0.0007	HOMO −3 → LUMO	96.39
7	S1	645.86	0.3025	HOMO → LUMO	99.01
	S2	566.14	0.0626	HOMO −1→ LUMO	98.57
	S3	506.92	0.0000	HOMO −2 → LUMO	99.39
8	S1	666.83	0.2811	HOMO → LUMO	99.58
	S2	573.16	0.0390	HOMO −1 → LUMO	99.30
	S3	528.62	0.0000	HOMO −2 → LUMO	99.41
9	S1	646.49	0.2849	HOMO → LUMO	99.61
	S2	554.49	0.0328	HOMO −1 → LUMO	99.37
	S3	525.37	0.0000	HOMO −2 → LUMO	99.39

Each modification by the electronic method is π–π * and includes all-atomic subunits. The most excellent oscillator strength (*f*) is due to the electronic development from S_0_ to S_1_. The S_1_ energy is just an electron exchange from the HOMO to the LUMO. Like the qualities of the oscillator, frequencies are progressively increased as formation lengths are increased due to electronic progression to S_0_ to S_1_. The transition from HOMO to LUMO is significant in S_0_ to S_1_, and, as may appear, the HOMO to LUMO decreases in the above exam. Spectra have a similar profile for all mixtures, containing a whole solid band for the higher energies somewhere in the range of 470 and 654 nm for the gas and 553–646 nm in chloroform, respectively. The determined frequency (*λ*_*max*_) of the contemplated intensifies diminishes in the accompanying request MMCB-TDP > CB-MMSDP > CB-MMODP > MMCB-TCP > MMCB-SCP > MMCB-BCO > MMCB-OCP > MMCB-BCT> MMCB-BCS, which is a similar request of the bandgap in the gas stage and almost similar trend follows in solvent phase. Such findings demonstrate that for all D–A monomers, only one band on a plausible locale (*λ*_*max*_ >500 nm) (Figures 7) can be made; with the wider frequency that will further acquire the skills of photographing to-elector transformation of the compared sun driven cells, CB-TDP, CB-SDP and CB-ODP will retain all the greater lights.

## 4. Conclusions

A quantum compound analysis on the mathematical and optoelectronic properties gained through DFT and TD-DFT/B3LYP/6-311 G was performed to illustrate D-A’s primary and optoelectronic qualities of monomers. The dihedral points from the carbazole to various meetings between the receivers. The results are harsh concerning the holding process and polymerization, ranging from 130° to 160° in gas and solvent. The dihedral points of 3,6-CB-ODP, CB-TDP, and CB-SDP are in a direction out of flat compared to the formation plane. Oxygen (O), sulfur (S), and selenium (Se) had different electron impacts. The CB-TDP and Copolymer Monomers’ quadruple second estimates are larger than those of CB monomers and are more grounded electron acceptors for sulphur (S) iota and selenium (Se). 3,6-MMCB-OCP ≈ 3,6-MMCB-BCO < 3,6-MMCB-SDP < 3,6-MMCB-SCP < 3,6-MMCB-TCP < 3,6-MMCB-TDP < 3,6-MMCB-BCS 3,6-MMCB-< BCT in both gas and solvent.

Subsequently, the optoelectrical properties of *E*_*HOMO*_,*E*_*LUMO*_,*Eg, E*_*opt*_, and *E*_*B*_ energies were critically updated. The findings also give the ability to influence the inborn optoelectronic properties of the comparative monomers by various acceptors, such as ODP, TDP, and SDP. The retention characteristics of UV–vis are obtained using the technique TD/DFT/B3LYP/6-311 G. Somewhere between 513 and 666 nm in the gas, intense assimilation has been achieved and dissolved. The estimates of the VOC of the atoms envisaged varying hypothetically from 0.80 to 1.3 eV. Finally, the results show how electronic properties can be tuned to a replacement with a few acceptors, recommending optoelectronic applications such as BHJ in solar cells of CB-ODP, CB-TDP and CB-SDP compounds.
